# Endoscopic Reflux Esophagitis and Reflux-Related Symptoms after *Helicobacter pylori* Eradication Therapy: Meta-Analysis

**DOI:** 10.3390/jcm9093007

**Published:** 2020-09-18

**Authors:** Mitsushige Sugimoto, Masaki Murata, Hitomi Mizuno, Eri Iwata, Naoyoshi Nagata, Takao Itoi, Takashi Kawai

**Affiliations:** 1 Department of Gastroenterological Endoscopy, Tokyo Medical University Hospital, Shinjuku, Tokyo 160-0023, Japan; eiwata@tokyo-med.ac.jp (E.I.); nnagata_ncgm@yahoo.co.jp (N.N.); t-kawai@tokyo-med.ac.jp (T.K.); 2 Department of Gastroenterology, National Hospital Organization Kyoto Medical Center, Kyoto 612-8555, Japan; mura05310531@gmail.com; 3 Toyoda Aoba Clinic, Iwata, Shizuoka 438-0821, Japan; hito0215@toyodaaobaclinic.com; 4 Department of Gastroenterology and Hepatology, Tokyo Medical University Hospital, Shinjuku, Tokyo 160-0023, Japan; itoitakao@gmail.com

**Keywords:** reflux esophagitis, *Helicobacter pylori*, eradication therapy, GERD, Western population

## Abstract

**Backgrounds:** The etiology of gastroesophageal reflux disease (GERD) including reflux esophagitis and non-erosive reflux disease is multifactorial and a recent meta-analysis showed no association between the development of GERD and *Helicobacter pylori* eradication in both Western and East-Asian populations. However, the problem remains that various inclusion criteria are used in these studies, which hinders meta-analysis. With a focus on reflux esophagitis with endoscopic mucosal injury, we meta-analysed to evaluate the association between eradication and reflux esophagitis and symptoms using a clearly defined set of inclusion criteria. **Methods:** We conducted a meta-analysis of studies published up until March 2020, which compared the incidence of reflux esophagitis and symptoms between patients undergoing *H. pylori* eradication therapy in a randomized placebo-controlled trial (Category A); between patients with successful and failed eradication (Category B); and between patients with successful vs. failed eradication, receipt of placebo, or no-treatment *H. pylori*-positives (Category C). **Results:** A total of 27 studies were included. Significant statistical effects were found for development of endoscopic reflux esophagitis [relative risk (RR): 1.46, 95% confidence interval (CI): 1.16–1.84, *p* = 0.01] or *de novo* reflux esophagitis (RR: 1.42, 95% CI: 1.01–2.00, *p* = 0.03) in the case group that received eradication in all studies, especially in Western populations. There was no significant difference in the incidence of symptoms after eradication between patient and control groups, regardless of category, location of population, or baseline disease. **Conclusions:** Eradication therapy for *H pylori* increases the risk of reflux esophagitis, irrespective of past history of esophagitis. In contrast, no effect was seen on reflux-related symptoms.

## 1. Introduction

Gastroesophageal reflux disease (GERD) is defined as the presence of acid-reflux-related symptoms, or endoscopic esophageal mucosal damage, caused by the abnormal reflux of gastric contents into the esophagus. GERD includes reflux esophagitis with endoscopically diagnosed mucosal damage, irrespective of the presence of symptoms and non-erosive reflux disease (NERD). The prevalence of GERD with reflux esophagitis and NERD has been increasing in East Asian countries, including Japan, since the end of the 20th century due to changes in environmental factors [1,2]. In general, the etiology of GERD is multifactorial and includes frequent and prolonged reflux of gastric contents, status and size of hiatal hernia, dysfunction of lower esophageal sphincter (LES), dysfunction of esophageal motility, hypersensitivity, and *H. pylori* infection. Both the increase in acid secretion and decrease in LES pressure play important roles in the development of reflux esophagitis. Understanding the pathophysiology of GERD and reflux esophagitis in individual patients is therefore an important aspect of treatment.

The Maastricht V/Florence consensus report recommends eradication therapy for *H pylori* infection for patients with peptic ulcer, gastric mucosa associated-lymphoid tissue lymphoma, atrophic gastritis, autoimmune thrombocytopenia, iron deficiency anemia, chronic urticaria, functional dyspepsia, and reflux esophagitis [3]. Considering the findings of systematic reviews and meta-analysis showing that *H. pylori* infection is inversely associated with GERD and reflux esophagitis [4,5,6], eradication therapy is also considered to increase the risk of GERD and reflux esophagitis and development of reflux-related symptoms. Many studies have evaluated the effects of *H. pylori* eradication on the development of GERD and reflux esophagitis and reflux-related symptoms, but results have been inconsistent and inconclusive [7,8,9,10,11,12,13,14,15,16,17,18,19,20,21,22,23,24,25,26,27,28,29,30,31,32,33]. In their meta-analysis, Xie et al. [34] reported a significantly increased risk of GERD in cohort studies of patients with successful eradication compared to those in whom eradication failed [risk ratio (RR): 1.70, 95% confidence interval (CI): 1.30–2.23] and a significantly increased risk in patients receiving eradication therapy compared with those receiving placebo (RR: 1.99, 95% CI: 1.23–3.22) in randomized control studies (RCT). However, most meta-analyses have reported no significant differences in the development of GERD following *H. pylori* eradication between patients with eradication and those with persistent infection, regardless of follow-up period, location (e.g., Western and East Asian populations), or baseline disease (e.g., peptic ulcer, functional dyspepsia, reflux esophagitis, and GERD) [35,36,37,38,39]. However, these meta-analyses are hindered by the inconsistencies among inclusion criteria used by the various studies. These inconsistencies involve variations in outcome measures (e.g., development of GERD, NERD, reflux esophagitis, and reflux-related symptoms), variations in case status (e.g., patients receiving eradication therapy and patients with successful eradication), variations in controls (e.g., patients receiving placebo, patients with failed eradication, and age- and disease-matched patients), and variations in study design (e.g., RCT, cohort study, and retrospective observational study). A conclusive evaluation of the associations between eradication therapy and the development of reflux esophagitis, not GERD and NERD, and reflux-related symptoms therefore requires a clearly defined set of patients and controls under the same study design.

Here, with a focus on endoscopically diagnosed reflux esophagitis of GERD, we performed a meta-analysis to compare the incidences of endoscopic reflux esophagitis, *de novo* reflux esophagitis, and reflux-related symptoms by dividing studies into three categories by study, setting of cases, and controls.

## 2. Materials and Methods

### 2.1. Search Strategy and Inclusion Criteria

**Figure 1:**
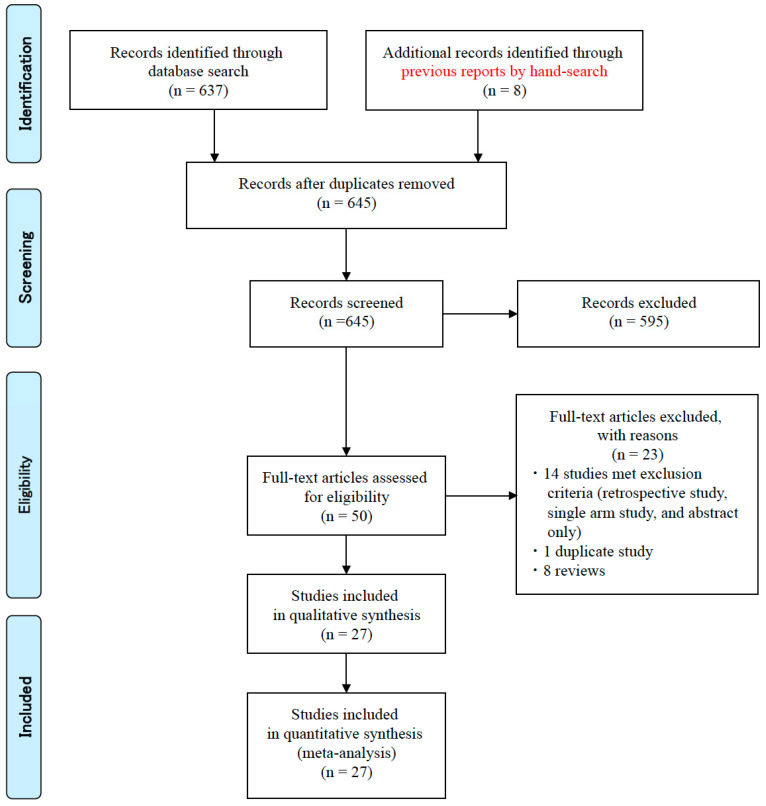
Workflow for the selection of studies comparing incidence rates of endoscopic reflux esophagitis and reflux-related symptoms.

Three researchers (M.S., M.M., and H.M.) independently searched both the PubMed and Cochrane Library databases using the terms “esophagitis”, “GERD”, “*Helicobacter pylori*,” and “eradication” and reviewed the titles and abstracts of all studies identified (Figure 1). The inclusion criteria were (1) RCTs or prospective cohort studies written in English published up to March 2020; (2) studies that compared incidence rates of endoscopic reflux esophagitis or reflux-related symptoms after *H. pylori* eradication therapy; (3) studies checking outcomes ≥4 weeks after eradication therapy; and (4) studies where the development of reflux esophagitis was endoscopically evaluated. Exclusion criteria were (1) studies performed under a retrospective design; (2) single-arm studies; (3) duplicated studies and multiple reports of the same study; and (4) studies with an abstract only. The full texts of candidate studies were then screened to select studies meeting the inclusion criteria. When multiple articles were found, we used data from that with the latest publication date.

We divided the studies into three categories: Category A were studies that compared the incidence rates of reflux esophagitis and reflux-related symptoms between patients receiving *H. pylori* eradication therapy (case) or placebo (control); Category B were studies that compared patients with successful eradication (case) or eradication failure and receipt of placebo (control, infection persisted); and Category C were studies that compared patients with successful eradication therapy (case) or eradication failure, receipt of placebo, and no-receipt of drug/placebo (control, infection persisted) [7,8,9,10,11,12,13,14,15,16,17,18,19,20,21,22,23,24,25,26,27,28,29,30,31,32,33]. Author names, publication year, country where the study was conducted, follow-up period, number of patients, smoking habit, alcohol use, sex, age, eradication regimen, eradication rate, and incidence rates of endoscopic reflux esophagitis and reflux-related symptoms, such as heart burn, discomfort, and chest pain, before and after treatment were extracted from each study.

### 2.2. Statistical Analysis

First, a meta-analysis of RCTs and cohort studies comparing incidence rates of reflux esophagitis, *de novo* reflux esophagitis, and reflux-related symptoms of the case versus control group was performed for each of the three categories. RRs and their corresponding 95% CIs were used to summarize the effect of each comparison tested using random-effects models and the calculated results were confirmed in a fixed-effects model as well [40,41,42]. Potential study bias in each study was evaluated by funnel plot tests. Heterogeneity was evaluated by the *I*^2^ value and Cochran’s Q. The *I*^2^ value was used to assess the heterogeneity of the studies as follows: 0–39%, low heterogeneity; 40–74%, moderate heterogeneity; and 75–100%, high heterogeneity.

All meta-analyses were conducted using open-source statistical software (Review Manager Version 5.3., The Nordic Cochrane Centre, The Cochrane Collaboration, Copenhagen, Denmark, 2014). All *p*-values were two-sided and *p* < 0.05 was considered statistically significant. Calculations were performed using commercial software (SPSS version 20, IBM Inc., Armonk, NY, USA).

## 3. Results

### 3.1. Literature Search and Data Extraction

The search strategy yielded 637 potentially eligible studies from the PubMed and Cochrane Library databases and eight studies by hand-search through other papers and meta-analysis (Figure 1). On review of titles and abstracts for all potential studies, 50 studies were selected from 645 extracted studies. Of these, 15 studies met the exclusion criteria (retrospective study design, single arm study, abstract only, and duplicated study) and eight were reviews, which were excluded. Finally, 27 full articles were assessed for eligibility (Figure 1) [7,8,9,10,11,12,13,14,15,16,17,18,19,20,21,22,23,24,25,26,27,28,29,30,31,32,33]. On categorization, 12 studies were assigned to Category A [7,8,9,10,11,12,13,14,15,16,17,18], 10 to Category B [19,20,21,22,23,24,25,26,27,28], and 5 to Category C [29,30,31,32,33].

Eradication rates for patients receiving eradication therapy in Category A varied (44.5–100%, mean eradication rate: 77.1% (1492/1933)). In contrast, eradication rates for the control groups receiving placebo in Category A were 0% to 14.0% (Table 1). In Categories B and C, although all case patients achieved eradication, eradication rates for controls at the end of the studies were unknown (Table 1 and Table 2). Regarding baseline disease, seven studies (three in Category A, one in Category B, and three in Category C) investigated the recurrence of GERD and symptoms after eradication therapy in patients with GERD and reflux esophagitis (Table 1). Eight studies evaluated East Asian populations, 17 studies evaluated Western populations, and two studies were conducted in Brazil.

### 3.2. Meta-Analysis for Incidence Rate of Reflux Esophagitis, de novo Esophagitis, and Symptoms

**Figure 2:**
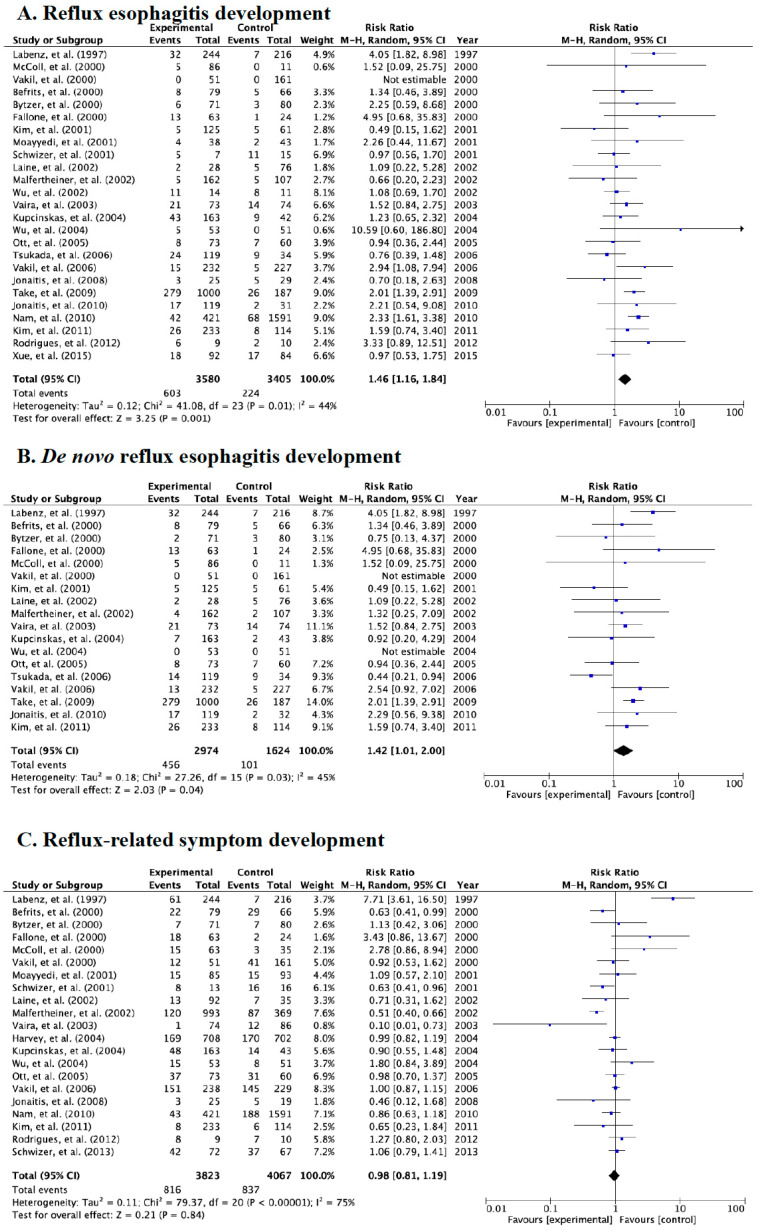
Forest plots of the rate of patients with reflux esophagitis development after eradication irrespective of whether they were free from gastroesophageal reflux disease (GERD) at baseline (**A**), the rate of *de novo* reflux esophagitis newly developed after eradication (**B**) and the rate of patients with reflux-related symptoms after eradication therapy (**C**) in a random-effects model. Significant differences in reflux esophagitis and *de novo* esophagitis were shown between patients receiving eradication therapy and those receiving placebo. Abbreviations: CI, confidence interval.

Of the 27 studies that investigated the development of endoscopically diagnosed reflux esophagitis and symptoms after eradication, 25 and 18 studies evaluated the incidence of reflux esophagitis and *de novo* reflux esophagitis, respectively (Table 2 and Figure 2A,B). When we combined all studies for meta-analysis, the overall incidence rates of reflux esophagitis and *de novo* esophagitis in the case group were 16.8% (603/3580, control group: 6.6%, 224/3405) and 15.3% (456/2974, control group: 6.2%, 101/1624), respectively. In addition, incidence rates of reflux-related symptoms were 21.3% (816/3823) in the case group and 20.6% (837/4067) in the control group after eradication therapy (Figure 2C).

Compared to the control group, a significant statistical effect was found for the development of endoscopic reflux esophagitis irrespective of whether they were free from GERD at baseline (relative risk (RR): 1.46, 95% CI: 1.16–1.840, *p* = 0.01) or endoscopic *de novo* reflux esophagitis (RR: 1.42, 95% CI: 1.01–2.00, *p* = 0.03) in the case group in all studies (Figure 2A,B).

However, we saw no significant difference in reflux-related symptoms after eradication between the case and control groups in the random-effects model (Figure 2C).

### 3.3. Meta-Analysis for the Incidence Rate of Reflux Esophagitis and Symptoms in Category A

**Figure 3:**
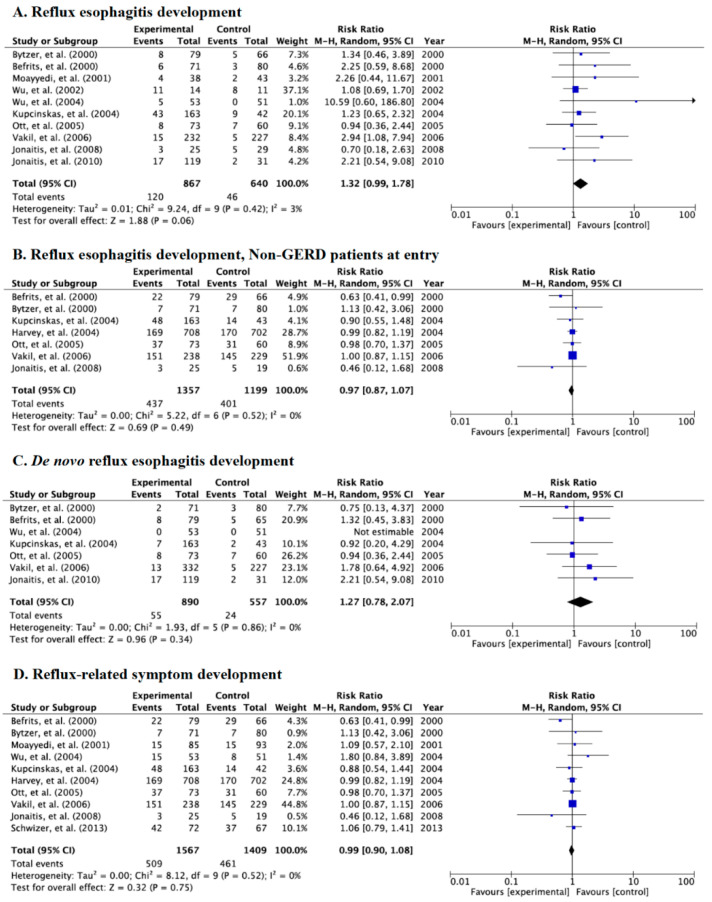
Forest plots of the rate of patients with reflux esophagitis development after eradication irrespective of whether they were free from gastroesophageal reflux disease (GERD) at baseline (**A**), reflux esophagitis after eradication in patients that were free from GERD at baseline (**B**), the rate of *de novo* reflux esophagitis newly developed after eradication (**C**), and the rate of patients with reflux-related symptoms after eradication therapy (**D**) in the random-effects model in Category A. No significant difference in reflux esophagitis and symptoms was shown between the case and control groups. Abbreviations: CI, confidence interval.

Of 12 RCTs in Category A, 10 studies endoscopically evaluated the incidence of reflux esophagitis, eight evaluated *de novo* reflux esophagitis, and 10 evaluated reflux-related symptoms (Table 2 and Figure 3A–D). There was no significant difference in the incidence of endoscopic reflux esophagitis among all RCTs (RR: 1.32, 95% CI: 0.99–1.78) (Figure 3A), in endoscopic reflux esophagitis in seven studies using patients free from GERD at baseline (RR: 0.97, 95% CI: 0.12–1.68) (Figure 3B), endoscopic *de novo* esophagitis (RR: 1.27, 95% CI: 0.78–2.07) (Figure 3C), and reflux-related symptoms (RR: 0.99, 95% CI: 0.90–1.08) (Figure 3D) between patients undergoing eradication and controls receiving placebo in the random-effects model. Test of heterogeneity was not significant for the meta-analysis (Figure 3A: *p* = 0.42, *χ*^2^ = 9.24, *I*^2^ = 3%, Figure 3B: *p* = 0.52, *χ*^2^ = 5.22, *I*^2^ = 0%, Figure 3C: *p* = 0.86, *χ*^2^ = 1.93, *I*^2^ = 0%, and Figure 3D: *p* = 0.52, *χ*^2^ = 8.12, *I*^2^ = 0%).

### 3.4. Meta-Analysis for Incidence Rate of Reflux Esophagitis and Symptoms in Category B

**Figure 4:**
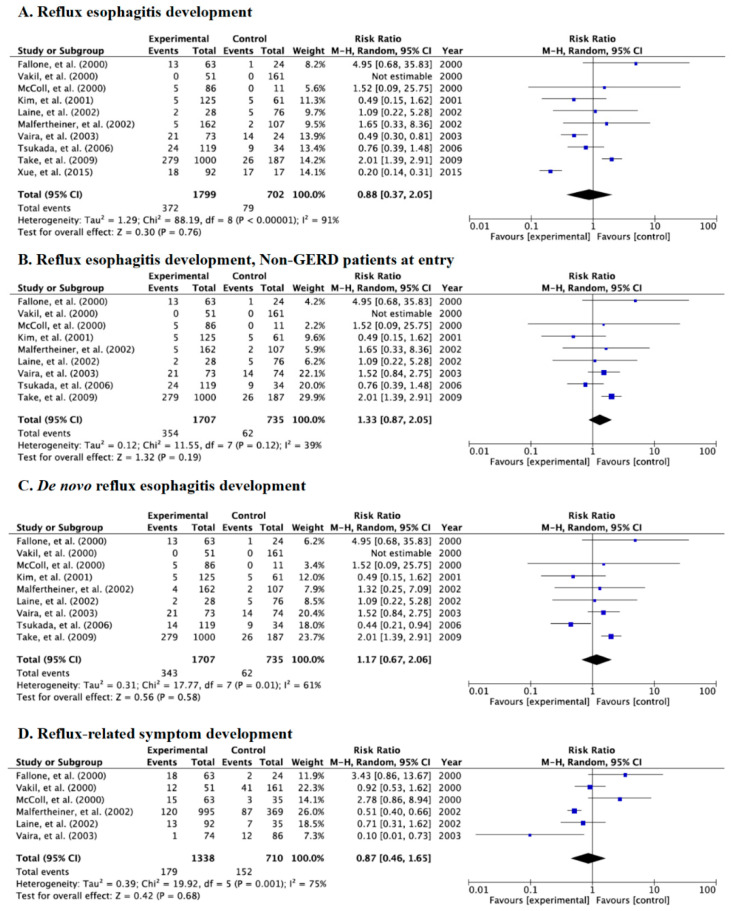
Forest plots of the rate of patients with reflux esophagitis development after eradication irrespective of whether they were free from gastroesophageal reflux disease (GERD) at baseline (**A**), reflux esophagitis after eradication in patients free from GERD at baseline (**B**), the rate of *de novo* reflux esophagitis newly developed after eradication (**C**), and the rate of patients with reflux-related symptoms after eradication therapy (**D**) in the random-effects model in Category B. No significant difference in reflux esophagitis and symptoms was shown between the case and control groups. Abbreviations: CI, confidence interval.

Category B including RCTs and prospective cohort studies investigated the incidence rates of endoscopic reflux esophagitis and symptoms between patients with successful eradication and those with failed eradication and receipt of placebo (control, infection persisted). Of 10 studies in Category B, all studies evaluated incidence of endoscopic reflux esophagitis and endoscopic *de novo* reflux esophagitis and six evaluated symptoms (Table 2 and Figure 4A–D). There were no significant differences in the incidence of endoscopic reflux esophagitis (RR: 0.88, 95% CI: 0.37–2.05) (Figure 4A); esophagitis in studies using patients free from GERD at baseline (RR: 1.33, 95% CI: 0.87–2.05) (Figure 4B); de novo esophagitis (RR: 1.17, 95% CI: 0.67–2.06) (Figure 4C); or symptoms (RR: 0.87, 95% CI: 0.46–1.65) (Figure 4D) in the random-effects model.

### 3.5. Meta-Analysis for Incidence Rate of Reflux Esophagitis and Symptoms in Category C

**Figure 5:**
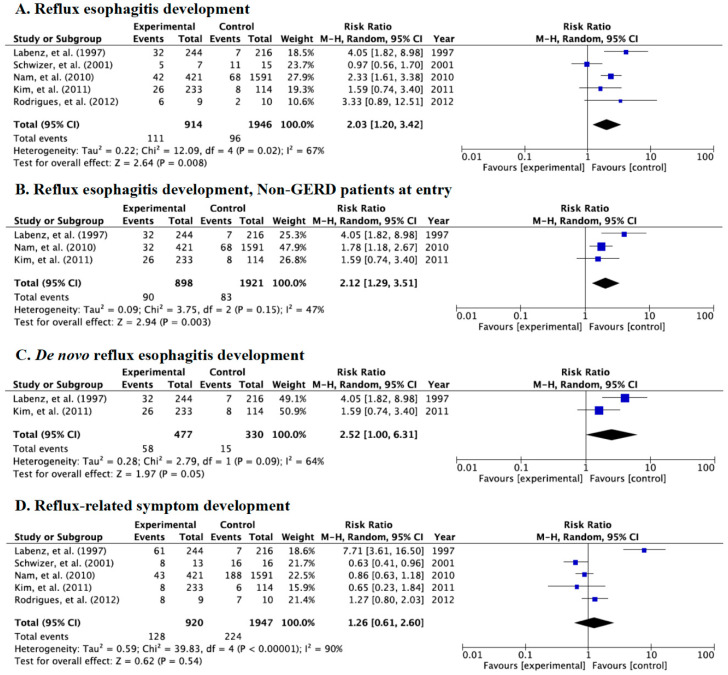
Forest plots of the rate of patients with reflux esophagitis development after eradication irrespective of whether they were free from gastroesophageal reflux disease (GERD) at baseline (**A**), reflux esophagitis after eradication in patients free from GERD at baseline (**B**), the rate of *de novo* reflux esophagitis newly developed after eradication (**C**), and the rate of patients with reflux-related symptoms after eradication therapy (**D**) in the random-effects model in Category C. Significant differences in reflux esophagitis and *de novo* esophagitis were shown between patients with successful eradication and the control group. Abbreviations: CI, confidence interval.

Category C was used to investigate the incidence rates of endoscopically diagnosed reflux esophagitis and symptoms between patients with successful eradication (case group) and those with eradication failure, receipt of placebo, and no-receipt of drug/placebo (control group, infection persisted). Significant differences between the case and control groups were shown in the incidences of endoscopic reflux esophagitis (RR: 2.03, 95% CI: 1.20–3.42) (Figure 5A), endoscopic esophagitis in studies using patients free from GERD at baseline (RR: 2.12, 95% CI: 1.29–3.51) (Figure 5B), and endoscopic *de novo* reflux esophagitis (RR: 2.52, 95% CI: 1.00–6.31) (Figure 5C) in the random-effects model.

There was no significant difference in reflux-related symptoms after eradication therapy (RR: 1.26, 95% CI: 0.61–2.60) (Figure 5D).

### 3.6. Meta-Analysis for the Incidence Rate of de novo Reflux Esophagitis and Symptoms between Western and East Asian Populations

**Figure 6:**
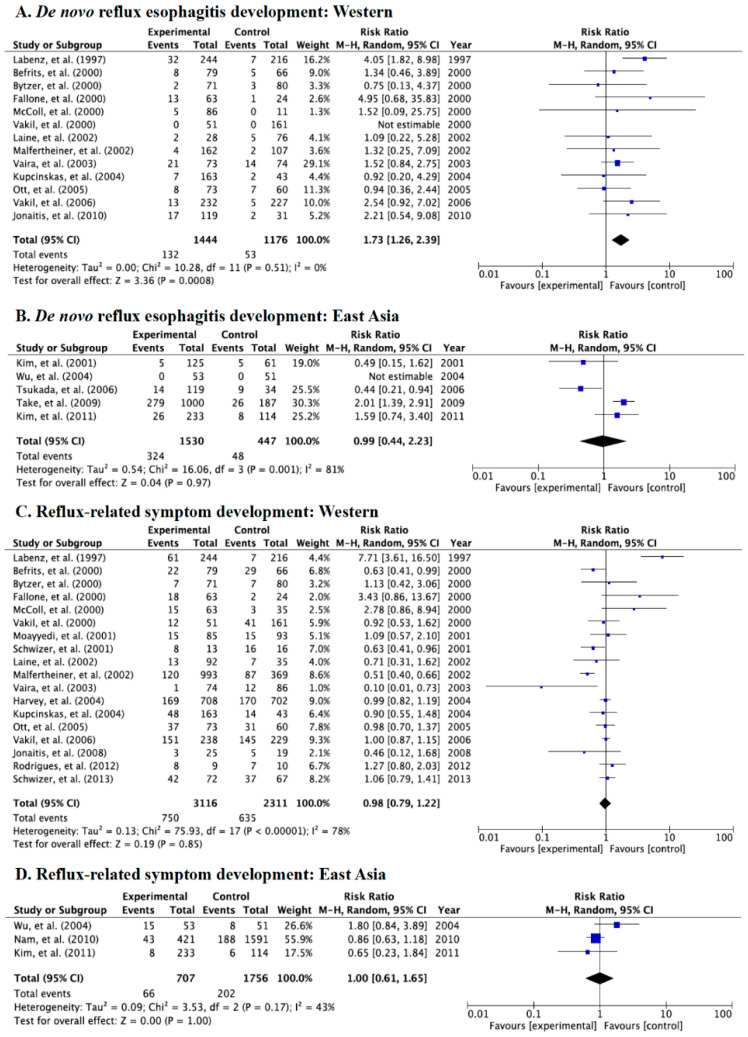
Forest plots of the rate of *de novo* reflux esophagitis newly developed after eradication in Western populations (**A**) and East Asian populations (**B**) and the rate of patients with reflux-related symptoms after eradication therapy in Western populations (**C**) and in East Asian populations (**D**). Significant differences are seen in the incidence of *de novo* reflux esophagitis in Western populations, whereas no significant differences are seen in East Asian populations. Abbreviations: CI, confidence interval.

We divided the studies into two different populations, Western (North and South America and Europe) and East Asian populations (Japan, China, and Korea). Overall, the incidence rates of *de novo* esophagitis in Western and East Asian populations in the case group were 9.1% (132/1444, the control group: 4.5%, 53/1176) and 21.2% (324/1530, the control group: 10.7%, 48/447), respectively. Significant differences were shown in the incidence of endoscopic *de novo* reflux esophagitis in Western populations (RR: 1.73, 95% CI: 1.26–2.39) (Figure 6A), but no significant difference was shown in East Asian populations (RR: 0.99, 95% CI: 0.44–2.23) (Figure 6B).

Incidence rates of reflux-related symptoms after eradication in Western and East Asian populations in the case group were 24.1% (750/3116, the control group: 27.5%, 635/2311) and 9.3% (66/707, the control group: 11.5%, 202/1756), respectively. There were no significant differences in the incidence of symptoms in both Western and East Asian populations (Figure 6C,D).

## 4. Discussion

This meta-analysis of 27 studies evaluated the incidence rates of endoscopic reflux esophagitis and acid-related symptoms after *H pylori* eradication therapy with categorization of studies into three kinds by study design. In the overall analysis, the incidence rates of endoscopic reflux esophagitis after eradication, including *de novo* reflux esophagitis, and reflux-related symptoms in studies using patients free of GERD at baseline were around 15% and 20%, respectively, irrespective of study design. In this meta-analysis, eradication therapy was associated with an increased risk of endoscopic reflux esophagitis development. Interestingly, the post-eradication risk of endoscopic reflux esophagitis and *de novo* esophagitis differed between Western and East Asian populations. Although patients with *H. pylori*-positive pyloric-predominant gastritis, such as Western populations, are hypothesized to experience inhibition of acid secretion after eradication therapy, this meta-analysis showed that Western populations had a higher risk of reflux esophagitis after eradication. The differences between our present and previous meta-analyses are likely due to our clear definition of the patient and control groups using the same study design [5,34]. Clarification of the characteristics of patients who develop endoscopic reflux esophagitis following *H. pylori* eradication therapy among Western populations is required.

### 4.1. Acid secretion after H. pylori Eradication Therapy

In general, although *H. pylori*-negative individuals without gastric mucosal inflammation and atrophy have a highly acidic intragastric pH of 1–2, acid secretion in *H. pylori*-positive patients differs by age and severity of inflammation and atrophy [43,44]. Infection with *H. pylori* in childhood results in gastric inflammation. Over time, the area of inflammation extends from the antrum to the body and finally, the ability to secrete acid decreases through the progressive atrophy-induced loss of acid-producing cells [45]. In addition, acid secretion is related with the infiltration of activated inflammatory cells that secrete pro-inflammatory cytokines [45,46]. When pyloric gastritis is predominant, IL-8 primarily stimulates gastrin-producing cells in the pyloric mucosa, resulting in hypergastrinemia and a consequent increase in acid secretion. Patients with pyloric-predominant gastritis are therefore at higher risk of duodenal ulcer and likely, also reflux esophagitis. The effects of TNF-alpha and IL-1beta are mainly observed after the extension of atrophy to the body. IL-1beta inhibits acid secretion with 100-fold greater potency than proton pump inhibitors (PPIs) on a molar basis [47]. Therefore, when body gastritis becomes dominant, acid secretion is substantially suppressed [45]. When this stage is reached, *H. pylori*-infected patients are at an increased risk of gastric ulcers and cancer, while the risk of reflux esophagitis decreases.

Eradication leads to the resolution of inflammation in the gastric fundic mucosa. The recovery of acid secretion that follows this resolution has led to concerns about the development of reflux esophagitis and symptoms [34]. In such patients with pyloric-predominant gastritis and potent acid secretion, eradication reduces gastrin stimulation by IL-8 and normalizes acid secretion, which is expected to prevent reflux esophagitis [48]. Koike et al. [49] reported the ability to secrete acid as well as intragastric pH change following eradication and identified the development of reflux esophagitis in patients with a substantial recovery in acid secretion after eradication. These findings indicate that the degree of gastritis at the time of eradication therapy influences the recovery of acid secretion and the subsequent risk of reflux esophagitis after eradication.

### 4.2. Development of Reflux Esophagitis after Eradication Therapy

Although many studies have investigated the development of GERD and/or endoscopic reflux esophagitis after eradication, including RCTs, prospective cohort studies, and retrospective observational case-control studies, no conclusive results have yet been obtained [7,8,9,10,11,12,13,14,15,16,17,18,19,20,21,22,23,24,25,26,27,28,29,30,31,32,33]. In 2003, Cremonini et al. [5] reported that pooled odds ratios for the development of GERD with inclusion of endoscopic reflex esophagitis and NERD, *de novo* GERD, and rebound/exacerbated GERD after eradication therapy were significantly increased in the case group (OR: 2.54, 95% CI: 1.92–3.37, OR: 3.25, 95% CI: 2.09–5.33 and OR: 2.39, 95% CI 1.75–3.34, respectively), while in 2013, Xie et al. [34] reported a significantly increased risk of GERD in patients with successful eradication compared with patients with eradication failure [RR: 1.70, 95% CI: 1.30–2.23] in a meta-analysis using a cohort study and a significantly increased risk in patients undergoing active eradication compared with those receiving placebo (RR: 1.99, 95% CI: 1.23–3.22) in a meta-analysis of RCTs. In contrast, another five meta-analyses failed to show a significant difference in the development of GERD after *H. pylori* eradication [35,36,37,38,39]. Recent evidence therefore suggests that there is no significant association between eradication therapy and development of GERD, and however, no evidence for an association with endoscopically diagnosed erosive esophagitis directly related with acid reflux to the esophagus. Because previous meta-analyses might not have been conducted with unification by study design (e.g., RCT, prospective cohort studies, and case-control studies), baseline disease (e.g., peptic ulcer, functional dyspepsia, reflux esophagitis, and GERD), outcome (e.g., development of GERD, reflux esophagitis and reflux-related symptoms) and location (e.g., Western and East Asian populations), the possibility of error is present. In addition, because pathogenesis of GERD and reflux esophagitis differs, in this study we focused on investigating the association of endoscopic reflux esophagitis and eradication therapy based on categorization by study design.

In this study, the incidence of endoscopic reflux esophagitis after eradication in the case group was around 15%, and significant effects were found for the development of reflux esophagitis (RR: 1.46, 95% CI: 1.16–1.840) and *de novo* esophagitis (RR: 1.42, 95% CI: 1.01–2.00). When we divided studies into three categories by study design, because sample size in each category will decrease, statistical analysis weakens. Although different results for Categories B and C are shown, significant risk of reflux esophagitis was shown in the incidence of reflux esophagitis; of reflux esophagitis in studies using patients free from GERD at baseline; and of *de novo* reflux esophagitis in the random-effects model in Category C. We therefore consider that if patients and controls are clearly categorized under the same type of study design, the risk of endoscopic reflux esophagitis after eradication therapy will be shown to increase. In contrast, no significant association will be seen between eradication and the development of GERD, as shown in previous meta-analyses [35,36,37,38,39].

### 4.3. Difference of Risk of Reflux Esophagitis after Eradication Therapy between East Asian and Western Populations

*H. pylori* infection is a protective factor for GERD and endoscopic reflux esophagitis [21,29,50]. Although the incidence rate of GERD and reflux esophagitis differs between East Asian and Western populations, this observation is attributable to differences in lifestyle, genetic factors, and virulence of *H. pylori* strains [51]. In fact, infection with *H. pylori* strains with high virulence factors (e.g., *oipA, dupA,* *cagA* and *vacA* s1m1) induces severe gastric mucosal inflammation with hypochlorhydria, increases in the risk of severe atrophy, peptic ulcer and gastric cancer. Indeed, most *H. pylori* strains seen in East Asian populations are *cagA*-positive and *vacA* s1m1-type *H. pylori* with high virulence [51]. The risk of reflux esophagitis after eradication might therefore differ between East Asian and Western populations. In fact, Cremonini et al. [5] reported that GERD development after eradication in East Asian populations was significantly higher than that in Western populations, while Xie et al. [34] reported a significantly increased risk of GERD in patients with successful eradication compared to those with eradication failure (RR: 4.53, 95% CI: 1.66–12.36) in a meta-analysis of Asian studies. However, these meta-analyses included case-control studies and single-arm non-control studies and investigated associations with the development of GERD, but not reflux esophagitis. In this meta-analysis, in contrast, we focused on reflux esophagitis. Although the overall incidence rate of *de novo* esophagitis in East Asian populations was 21.2%, which was higher than that in Western populations (9.1%) after eradication therapy, significant differences were shown in the incidence of *de novo* reflux esophagitis in Western populations (RR: 1.73, 95% CI: 1.26–2.39), but not in East Asian populations. This observation may suggest that the pathogenesis of GERD and reflux esophagitis differs and that eradication therapy increases the risk of GERD in East Asian populations. Patients considering eradication therapy should therefore be required to give carefully informed consent about the possibility of GERD development and appropriate administration of acid secretion inhibitors should be considered.

### 4.4. Development of Reflux-Related Symptoms after Eradication Therapy

In previous meta-analysis, no significant differences were observed in heartburn scores, healing, and relapse rates between *H. pylori* positives and negatives with endoscopic reflux esophagitis [39] and there was no significant difference in the rate of symptomatic GERD after eradication between patients with *H. pylori* eradicated and those with persistent infection, regardless of follow-up period, location, or the baseline [37]. Also, in this meta-analysis, we saw no significant difference in reflux-related symptoms between the case and control groups. However, in clinical practice, for any patients that experience reflux-related symptoms after eradication, it is required that the characteristics of patients who develop symptoms after eradication are clarified. Because *H. pylori* positive-patients have an increased risk of endoscopic reflux esophagitis, acid reflux is expected to increase, irrespective of the presence of reflux-related symptoms. In general, an association with endoscopic reflux esophagitis and reflux is reported.

## 5. Limitations

This meta-analysis has a few limitations. First, there is a possibility that selection bias exists because of the exclusion of the studies published in a language other than English, the unpublished studies, and the abstract alone. Second, because any studies that included patients received a PPI and H2RAs, this situation may influence the incidence of endoscopic reflux esophagitis and acid-related symptoms after eradication. We did not do the sub-analysis for the effects of endoscopic reflux esophagitis development by eradication on the PPI and/or H2RAs therapy. Third, there was a variety of eradication regimens, kinds and severity of acid-related symptoms, and healing time of endoscopic reflux esophagitis. Fourth, when we divided the studies into three categories, because the sample size in each category decreased, this weakened the statistical analysis. Future meta-analyses using many studies investigating the association between eradication and endoscopic reflux esophagitis should be reevaluated in a unified manner, with adjustment for background factors and evaluation of the outcomes.

## 6. Conclusions

In conclusion, since pharmacotherapy, including *H. pylori* eradication therapy, inevitably involves benefits as well as the risk of adverse effects, the advantages and disadvantages should be considered on a case-by-case basis and only undertaken if the advantages outweigh the disadvantages. Because *H. pylori* eradication treatment increases the risk of developing endoscopically diagnosed reflux esophagitis, particularly in Western populations, physicians should inform patients who received eradication treatment that they may be required to take medication, such as PPI.

## Figures and Tables

**Table 1:** Characteristics of the trials.

Authors (Year)	Country	Disease	Follow-up Period (months)	Number of Patients/Controls (*n*/*n*)	Patient Sex (M/F)	Patient Age (year)	Patient Smoking (*n*/*n*)	Patient Alcohol (*n*/*n*)	Outcome	Eradication Regimen	Eradication Rate (Patients) (%, (*n*/*n*))	Eradication Rate (Control) (%, (*n*/*n*))
Category A												
Befrits et al. (2000) [7]	Norway	DU	24	110/55	NA	NA	NA	NA	*de novo*	O (40)/A (750), 2 weeks	49/110 (44.5%)	1/55 (1.8%)
Bytzer et al. (2000) [8]	Denmark	DU	24	139/137	104/35	53.4 ± 13.0	67/139	NA	*de novo*	O (20)/A (750, t)/M (500, t), 2 weeks	84/139 (60.4%)	NA
Moayyedi et al. (2001) [9]	England	GERD	12	93/97	38/47	47.4 ± 12.5	27/85	56/85	*de novo*	O (20)/Tinidazole (500)/C (250), 1 week	70/85 (82.4%)	12/93 (12.9%)
Wu et al. (2002) [10]	Hong Kong	RR	4	14/11	9/5	51.3 ± 12.3	2/14	NA	Recurrent	O (20)/A (1000)/C (500), 1 week	14/14 (100%)	NA
Wu et al. (2004) [11]	Hong Kong	GERD	12	53/51	26/27	54.0 ± 13.8	7/53	10/53	Recurrent	O (20)/A (1000)/C (500), 1 week	52/53 (98.1%)	2/51 (3.9%)
Kupcinskas et al. (2004) [12]	Lithuania	DU	12	163/42	106/57	41.6 ± 13.2	73/163	NA	*de novo*	R (300)/A (1000)/M (400), 2 weeks or FAM (40)/A (1000)/M (400), 2 weeks, or O (20)/C (250)/M (400), 1 week, or O (20)/A (1000)/M (800), 1 week, or O (20)/A (1000)/C (500), 1 week	92/163 (56.4%)	NA
Harvey et al. (2004) [13]	England	Gastritis	24	787/771	385/402	NA	362/767	140/767	*de novo*	Ranitidine bismuth (400)/C (500), 2 weeks	659/727 (90.6%)	99/706 (14.0%)
Ott et al. (2005) [14]	Brazil	FD	12	82/75	18/64	41.5 ± 12.0	17/82	10/82	*de novo*	L (30)/A (1000)/C (500), 10 days	74/82 (90.2%)	1/75 (1.3%)
Vakil et al. (2006) [15]	Western	FD	12	297/306	116/181	49 ± 14	77/297	NA	*de novo*	O (20)/A (1000)/C (500), 1 week	243/297 (81.8%)	10/306 (3.7%)
Jonaitis et al. (2008) [16]	Lithuania	GU	12	54/34	27/17	51.3 ± 13.7	14/44	NA	*de novo*	O (20)/A (1000)/C (500), 1 week or O (20)/A (1000)/M (400), 1 week or ranitidine (300)/A (1000)/C (500), 2 weeks	25/44 (56.8%)	0/25 (0%)
Jonaitis et al. (2010) [17]	Lithuania	DU	12	119/31	NA	NA	NA	NA	*de novo*	O (20)/A (1000)/C (500), 1 week or O (20)/A (1000)/M (400), 1 week or ranitidine (300)/A (1000)/C (500), 2 weeks	70/119 (58.8%)	0/31 (0%)
Schwizer et al. (2013) [18]	Europe	GERD	2.7	100/98	NA	49 (20–75)	NA	NA	Recurrent	E (20)/A (1000)/C (500), 1 week	59/100 (59.0%)	NA
Category B												
Fallone et al. (2000) [19]	Canada	DU	12	63/34	45/18	48 ± 14	22/63	35/63	*de novo*	Bismuth/M/A or Bismuth/M or M	63/87 (72.4%)	
Vakil et al. (2000) [20]	USA	DU	12	64/178	56/8	49 ± 12	17/64	19/64	*de novo*	Ranitidine bismuth/C or Ranitidine bismuth/A	64/242 (26.4%)	
McColl et al. (2000) [21]	Scotland	PU	6	86/11	NA	NA	NA	NA	*de novo*	O (20)/M (400, t)/A (500, t) or O (20)/M (400, t)/TC (500, t), 2 weeks	70/97 (72.2%)	
Kim et al. (2001) [22]	Korea	PU	24	125/61	105/20	NA	75/125	79/125	*de novo*	O (20)/A (750)/C (200), 1–2 weeks	125/186 (67.2%)	
Malfertheiner et al. (2002) [23]	Germany	PU	6	369/993	NA	NA	NA	NA	*de novo*	O (20)/A (1000)/C (500) or O (20)/M (400)/C (250) or O (20)/A (1000)/M (400) or A (1000)/C (500) or M (400)/C (250), 7 days	369/1421 (26.9%)	
Laine et al. (2002) [24]	USA	DU	8	621/544	NA	NA	NA	NA	*de novo*	O (40)/A (500, t), 2 weeks or O (20)/A (1000, t), 2 weeks or O (20)/A (1000)/C (500), 10 days or E (40)/A (1000)/C (500), 10 days or E (40)/C (500), 10 days or E (40)/A (1000)/C (500), 10 days	621/1165 (53.3%)	
Vaira et al. (2003) [25]	Italy	Gastritis	102	81/88	56/25	47 ± 12	9/81	15/81	*de novo*	Unknown	81/169 (47.9%)	
Tsukada et al. (2006) [26]	Japan	PU	48	119/34	NA	NA	NA	NA	*de novo*	L (30)/A (750)/C (200 or 400), 1 week	119/163 (73.0%)	
Take et al. (2009) [27]	Japan	PU	43	1000/187	NA	NA	NA	NA	*de novo*	O (20)/A (750)/C (200 or 400) or L (30)/A (750)/C (200 or 400) or R (10)/A (750)/C (200 or 400), 1 week	NA	
Xue et al. (2015) [28]	China	RR	2	92/84	69/23	48.3 ± 13.0	23/69	16/76	Healing	E (20)/A (1000)/C (500), 1 week, or sequential regimen (E/A + E/C/tinidazole), 10 days	92/176 (52.3%)	
Category C												
Labenz et al. (1997) [29]	Germany	DU	17	244/216	155/92	52.9 ± 14.5	115/244	81/244	*de novo*	Unknown	244/460 (53.0%)	
Schwizer et al. (2001) [30]	Switzerland	GERD	6	13/16	2/11	54 ± 9	NA	NA	Recurrent	L (30)/A (1000)/C (500), 10 days	13/20 (65.0%)	
Nam et al. (2010) [31]	Korea	RR	24	465/1591	NA	NA	NA	NA	*de novo*	L (30)/A (1000)/C (500), 1 week	421/548 (76.8%)	
Kim et al. (2011) [32]	Korea	PU	24	233/114	NA	NA	NA	NA	*de novo*	1st, PPI/A (1000)/C (500), 1 week, 2nd, E (20)/bismuthate (300, q)/M (500, t)/TC (500, q), 1–2 weeks	233/347 (67.1%)	
Rodrigues et al. (2012) [33]	Brazil	GERD	3	9/10	6/3	37.4 ± 12.5	NA	3/9	*de novo*	L (30)/A (1000)/C (500), 1 week	9/11 (81.8%)	

Abbreviations: A; amoxicillin, C; clarithromycin, DU; duodenal ulcer, E; esomeprazole, F; female, FAM; famotidine, FD; functional dyspepsia, GERD; gastroesophageal reflux disease, GU; gastric ulcer, L; lansoprazole, M; male, NA; not available, O; omeprazole, PU; peptic ulcer, q; four-times-daily dosing, R; ranitidine, RR: reflux esophagitis, t; three-time-daily dosing, TC; tetracycline.

**Table 2:** Development of erosive esophagitis and reflux-related symptoms after *Helicobacter pylori* eradication therapy.

Authors (Year)	Number of Patients at Entry (*n*)		Number of Patients with RR at Entry (% (*n*/*n*))		Number of Patients with RR Development after Eradication (% (*n*/*n*))		Number of Patients with *de novo* RR Development after Eradication (*n*)		Number of Patients with Symptoms at Entry (% (*n*/*n*))		Number of Patients with Symptoms after Eradication (% (*n*/*n*))	
	Eradicated	Control	Eradicated	Control	Eradicated	Control	Eradicated	Control	Eradicated	Control	Eradicated	Control
Category A												
Befrits et al. [7]	94	51	9% (0/94)	0% (0/51)	10.1% (8/79)	7.6% (5/66)	10.1% (8/79)	7.6% (5/66)	NA	NA	27.8% (22/79)	43.9% (29/66)
Bytzer et al. [8]	139	137	7.2% (10/139)	5.8% (8/137)	8.5% (6/71)	3.8% (3/80)	2.8% (2/71)	3.8% (3/80)	28.1% (39/139)	25.5% (35/137)	9.9% (7/71)	8.8% (7/80)
Moayyedi et al. [9]	85	93	23.5% (20/85)	20.4% (19/93)	10.5% (4/38)	4.7% (2/43)	NA	NA	100% (85/85)	100% (93/93)	17.6% (15/85)	16.1% (15/93)
Wu et al. [10]	14	11	100% (14/14)	100% (11/11)	78.6% (11/14)	72.7% (8/11)	NA	NA	100% (14/14)	100% (11/11)	NA	NA
Wu et al. [11]	53	51	28.3% (15/53)	31.4% (16/51)	9.4% (5/53)	0% (0/51)	0% (0/53)	0% (0/51)	100% (53/53)	100% (51/51)	28.3% (15/53)	15.7% (8/51)
Kupcinskas et al. [12]	163	42	27.0% (44/163)	26.2% (11/42)	26.3% (43/163)	20.9% (9/43)	4.3% (7/163)	4.7% (2/43)	47.2% (77/163)	40.5% (17/42)	29.4% (48/163)	32.6% (14/43)
Harvey et al. [13]	708	702	NA	NA	NA	NA	NA	NA	53.4% (378/708)	52.4% (368/702)	23.9% (169/708)	24.2% (170/702)
Ott et al. [14]	82	75	0% (0/82)	0% (0/75)	11.0% (8/73)	11.7% (7/60)	11.0% (8/73)	11.7% (7/60)	54.9% (45/82)	48.0% (36/75)	50.7% (37/73)	51.7% (31/60)
Vakil et al. [15]	297	306	2.7% (8/297)	1.6% (5/306)	6.5% (15/232)	2.2% (5/227)	5/6% (13/232)	2.2% (5/227)	75.8% (225/297)	75.2% (230/306)	63.4% (151/238)	63.3% (145/229)
Jonaitis et al. [16]	44	25	18.2% (8/44)	16.0% (4/25)	12.0% (3/25)	17.2% (5/29)	NA	NA	18.2% (8/44)	16.0% (4/25)	12.0% (3/25)	26.3% (5/19)
Jonaitis et al. [17]	119	31	0% (0/119)	0% (0/31)	14.3% (17/119)	6.5% (2/31)	14.3% (17/119)	6.5% (2/31)	NA	NA	NA	NA
Schwizer et al. [18]	72	67	NA	NA	NA	NA	NA	NA	NA	NA	58.3% (42/72)	52.2% (37/67)
Category B												
Fallone et al. [19]	63	24	0% (0/63)	0% (0/24)	20.6% (13/63)	4.2% (1/24)	20.6% (13/63)	4.2% (1/24)	0% (0/63)	0% (0/24)	28.6% (18/63)	8.3% (2/24)
Vakil et al. [20]	64	178	0% (0/64)	0% (0/178)	0% (0/51)	0% (0/161)	0% (0/51)	0% (0/161)	57.8% (37/64)	51.7% (92/178)	23.5% (12/51)	25.5% (41/161)
McColl et al. [21]	86	11	0% (0/86)	0% (0/11)	5.8% (5/86)	0% (0/11)	5.8% (5/86)	0% (0/11)	0% (0/86)	0% (0/11)	23.8% (15/63)	8.6% (3/35)
Kim et al. [22]	125	61	0% (0/125)	0% (0/61)	4.0% (5/125)	8.2% (5/61)	4.0% (5/125)	8.2% (5/61)	NA	NA	NA	NA
Malfertheiner et al. [23]	369	993	0.6% (1/162)	0.9% (1/107)	3.1% (5/162)	1.9% (2/107)	2.5% (4/162)	1.9% (2/107)	33.5% (333/993)	40.7% (150/369)	12.1% (120/993)	23.6% (87/369)
Laine et al. [24]	621	544	0% (0/621)	0% (0/544)	7.1% (2/28)	6.6% (5/76)	7.1% (2/28)	6.6% (5/76)	0% (0/621)	0% (0/544)	14.1% (13/92)	20.0% (7/35)
Vaira et al. [25]	81	88	0% (0/81)	0% (0/88)	28.8% (21/73)	18.9% (14/74)	28.8% (21/73)	18.9% (14/74)	0% (0/81)	0% (0/88)	1.4% (1/74)	14.0% (12/86)
Tsukada et al. [26]	119	34	0% (0/119)	0% (0/34)	20.2% (24/119)	26.5% (9/34)	11.8% (14/119)	26.5% (9/34)	0% (0/119)	0% (0/34)	NA	NA
Take et al. [27]	1000	187	0% (0/1000)	0% (0/187)	27.9% (279/1000)	13.9% (26/187)	27.9% (279/1000)	13.9% (26/187)	NA	NA	NA	NA
Xue et al. [28]	92	84	100% (92/92)	100% (84/84)	19.6% (18/92)	20.2% (17/84)	NA	NA	100% (92/92)	100% (84/84)	NA	NA
Category C												
Labenz et al. [29]	244	216	0% (0/244)	0% (0/216)	13.1% (32/244)	3.2% (7/216)	13.1% (32/244)	3.2% (7/216)	29.9% (73/244)	NA	25.0% (61/244)	3.2% (7/216)
Schwizer et al. [30]	13	16	NA	NA	71.4% (5/7)	73.3% (11/15)	NA	NA	61.5% (8/13)	100% (16/16)	61.5% (8/13)	100% (16/16)
Nam et al. [31]	421	1591	4.0% (17/421)	2.9% (46/1591)	10.0% (42/421)	4.3% (68/1591)	NA	NA	6.7% (28/421)	6.2% (99/1591)	10/2% (43/421)	11.8% (188/1591)
Kim et al. [32]	233	114	0% (0/233)	0% (0/114)	11.2% (26/233)	7.0% (8/114)	11.2% (26/233)	7.0% (8/114)	0% (0/233)	0% (0/114)	3.4% (8/233)	5.3% (6/114)
Rodrigues et al. [33]	9	10	55.6% (5/9)	50% (5/10)	66.7% (6/9)	20.0% (2/10)	NA	NA	100% (9/9)	100$ (10/10)	88.9% (8/9)	70.0% (7/10)

## References

[1] Kinoshita Y., Kawanami C., Kishi K., Nakata H., Seino Y., Chiba T. (1997). *Helicobacter pylori* independent chronological change in gastric acid secretion in the Japanese. Gut.

[2] Fujiwara Y., Arakawa T. (2009). Epidemiology and clinical characteristics of GERD in the Japanese population. J. Gastroenterol..

[3] Malfertheiner P., Megraud F., O’Morain C., Gisbert J.P., Kuipers E.J., Axon A.T., Bazzoli F., Gasbarrini A., Atherton J., Graham D.Y. (2016). Management of *Helicobacter pylori* infection—The Maastricht V/Florence Consensus Report. Gut.

[4] Raghunath A., Hungin A.P.S., Wooff D., Childs S. (2003). Prevalence of *Helicobacter pylori* in patients with gastro-oesophageal reflux disease: Systematic review. Br. Med. J..

[5] Cremonini F., Di Caro S., Delgado-Aros S., Sepulveda A., Gasbarrini G., Camilleri M. (2003). Meta-analysis: The relationship between *Helicobacter pylori* infection and gastro-oesophageal reflux disease. Aliment. Pharmacol. Ther..

[6] Sugimoto M., Uotani T., Ichikawa H., Andoh A., Furuta T. (2016). Gastroesophageal Reflux Disease in Time Covering Eradication for All Patients Infected with *Helicobacter pylori* in Japan. Digestion.

[7] Befrits R., Sjöstedt S., Ödman B., Sörngård H., Lindberg G. (2000). Curing *Helicobacter pylori* infection in patients with duodenal ulcer does not provoke gastroesophageal reflux disease. Helicobacter.

[8] Bytzer C.A.P. (2000). Eradication of *Helicobacter pylori* Compared with Long-Term Acid Suppression in Duodenal Ulcer Disease: A Randomized Trial with 2-Year Follow-up. Scand. J. Gastroenterol..

[9] Moayyedi P., Bardhan C., Young L., Dixon M.F., Brown L., Axon A.T. (2001). *Helicobacter pylori* eradication does not exacerbate reflux symptoms in gastroesophageal reflux disease. Gastroenterology.

[10] Wu J.C.Y., Chan F.K., Wong S.K.H., Lee Y.T., Leung W.K., Sung J.J. (2002). Effect of *Helicobacter pylori* eradication on oesophageal acid exposure in patients with reflux oesophagitis. Aliment. Pharmacol. Ther..

[11] Wu J.C.Y., Chan F.K., Ching J.Y.L., Leung W.K., Hui Y., Leong R.W., Chung S.C.S., Sung J.J. (2004). Effect of *Helicobacter pylori* eradication on treatment of gastro-oesophageal reflux disease: A double blind, placebo controlled, randomised trial. Gut.

[12] Kupcinskas L., Jonaitis L., Kiudelis G. (2004). A 1 year follow-up study of the consequences of *Helicobacter pylori* eradication in duodenal ulcer patients. Eur. J. Gastroenterol. Hepatol..

[13] Harvey R.F., Lane J.A., Murray L.J., Harvey I.M., Donovan J.L., Nair P. (2004). Randomised controlled trial of effects of *Helicobacter pylori* infection and its eradication on heartburn and gastro-oesophageal reflux: Bristol helicobacter project. Br. Med. J..

[14] Ott E.A., Mazzoleni L.E., Edelweiss M.I., Sander G.B., Wortmann A.C., Theil A.L., Somm G., Cartell A., Rivero L.F., Uchôa D.M. (2005). *Helicobacter pylori* eradication does not cause reflux oesophagitis in functional dyspeptic patients: A randomized, investigator-blinded, placebo-controlled trial. Aliment. Pharmacol. Ther..

[15] Vakil N., Talley N.J., Stolte M., Sundin M., Junghard O., Bolling-Sternevald E. (2006). Patterns of gastritis and the effect of eradicating *Helicobacter pylori* on gastro-oesophageal reflux disease in Western patients with non-ulcer dyspepsia. Aliment. Pharmacol. Ther..

[16] Jonaitis L., Kiudelis G., Kupcinskas L. (2008). Gastroesophageal reflux disease after *Helicobacter pylori* eradication in gastric ulcer patients: A one-year follow-up study. Medicina.

[17] Jonaitis L., Kupčinskas J., Kiudelis G., Kupčinskas L. (2010). De novo erosive esophagitis in duodenal ulcer patients related to pre-existing reflux symptoms, smoking, and patient age, but not to *Helicobacter pylori* eradication: A one-year follow-up study. Medicina.

[18] Schwizer W., Menne D., Schütze K., Vieth M., Goergens R., Malfertheiner P., Leodolter A., Fried M., Fox M.R. (2013). The effect of *Helicobacter pylori* infection and eradication in patients with gastro-oesophageal reflux disease: A parallel-group, double-blind, placebo-controlled multicentre study. United Eur. Gastroenterol. J..

[19] Fallone C.A., Barkun A.N., Friedman G., Mayrand S., Loo V., Beech R., Best L., Joseph L. (2000). Is *Helicobacter pylori* eradication associated with gastroesophageal reflux disease?. Am. J. Gastroenterol..

[20] Vakil N., Hahn B., McSorley D. (2000). Recurrent symptoms and gastro-oesophageal reflux disease in patients with duodenal ulcer treated for *Helicobacter pylori* infection. Aliment. Pharmacol. Ther..

[21] McColl K.E., Dickson A., El-Nujumi A., El–Omar E.M., Kelman A. (2000). Symptomatic benefit 1-3 years after h. pylori eradication in ulcer patients: Impact of gastroesophageal reflux disease. Am. J. Gastroenterol..

[22] Kim N., Lim S.H., Lee K.H. (2001). No protective role of *Helicobacter pylori* in the pathogenesis of reflux esophagitis in patients with duodenal or benign gastric ulcer in Korea. Dig. Dis. Sci..

[23] Malfertheiner P., Dent J., Zeijlon L., Sipponen P., Van Zanten S.J.O.V., Burman C.-F., Lind T., Wrangstadh M., Bayerdörffer E., Lonovics J. (2002). Impact of *Helicobacter pylori* eradication on heartburn in patients with gastric or duodenal ulcer disease—Results from a randomized trial programme. Aliment. Pharmacol. Ther..

[24] Laine L., Sugg J. (2002). Effect of *Helicobacter pylori* eradication on development of erosive esophagitis and gastroesophageal reflux disease symptoms: A post hoc analysis of eight double blind prospective studies. Am. J. Gastroenterol..

[25] Vaira D., Vakil N., Rugge M., Gatta L., Ricci C., Menegatti M., Leandro G., Holton J., Russo V.M., Miglioli M. (2003). Effect of *Helicobacter pylori* eradication on development of dyspeptic and reflux disease in healthy asymptomatic subjects. Gut.

[26] Tsukada K., Katoh H., Miyazaki T., Fukuchi M., Kuwano H., Kimura H., Fukai Y., Inose T., Motojima T., Toda N. (2006). Factors Associated with the Development of Reflux Esophagitis After *Helicobacter pylori* Eradication. Dig. Dis. Sci..

[27] Take S., Mizuno M., Ishiki K., Nagahara Y., Yoshida T., Yokota K., Oguma K., Okada H., Yamamoto K. (2009). *Helicobacter pylori* eradication may induce de novo, but transient and mild, reflux esophagitis: Prospective endoscopic evaluation. J. Gastroenterol. Hepatol..

[28] Xue Y., Zhou L.-Y., Lin S.-R., Hou X.-H., Li Z.-S., Chen M.-H., Yan X.-E., Meng L.-M., Zhang J., Lü J.-J. (2015). Effect of *Helicobacter pylori* Eradication on Reflux Esophagitis Therapy. Chin. Med. J..

[29] Labenz J., Blum A.L., Bayerdörffer E., Meining A., Stolte M., Börsch G. (1997). Curing *Helicobacter pylori* infection in patients with duodenal ulcer may provoke reflux esophagitis. Gastroenterology.

[30] Schwizer W., Thumshirn M., Dent J., Guldenschuh I., Menne D., Cathomas G., Fried M. (2001). *Helicobacter pylori* and symptomatic relapse of gastro-oesophageal reflux disease: A randomised controlled trial. Lancet.

[31] Nam S.Y., Choi I.J., Ryu K.H., Kim B.C., Kim C.G., Nam B.-H. (2010). Effect of *Helicobacter pylori* Infection and Its Eradication on Reflux Esophagitis and Reflux Symptoms. Am. J. Gastroenterol..

[32] Kim N., Lee S.W., Kim J.I., Baik G.H., Kim S.J., Seo G.S., Oh H.J., Kim S.W., Jeong H., Hong S.J. (2011). Effect of *Helicobacter pylori* Eradication on the Development of Refl ux Esophagitis and Gastroesophageal Refl ux Symptoms: A Nationwide Multi-Center Prospective Study. Gut Liver.

[33] Rodrigues L., De Faria C.M., Geocze S., Chehter L. (2012). *Helicobacter pylori* eradication does not influence gastroesophageal reflux disease: A prospective, parallel, randomized, open-label, controlled trial. Arq. Gastroenterol..

[34] Xie T., Cui X.-B., Zheng H., Chen D., He L., Jiang B. (2013). Meta-analysis. Eur. J. Gastroenterol. Hepatol..

[35] Tan J., Wang Y., Sun X., Cui W., Ge J., Lin L. (2015). The Effect of *Helicobacter pylori* Eradication Therapy on the Development of Gastroesophageal Reflux Disease. Am. J. Med. Sci..

[36] Saad A.M., Choudhary A., Bechtold M.L. (2012). Effect of *Helicobacter pylor* itreatment on gastroesophageal reflux disease (GERD): Meta-analysis of randomized controlled trials. Scand. J. Gastroenterol..

[37] Qian B., Ma S., Shang L., Qian J., Zhang G. (2011). Effects of *Helicobacter pylori* Eradication on Gastroesophageal Reflux Disease. Helicobacter.

[38] Yaghoobi M., Farrokhyar F., Yuan Y., Hunt R.H. (2010). Is There an Increased Risk of GERD After *Helicobacter pylori* Eradication? A Meta-Analysis. Am. J. Gastroenterol..

[39] Raghunath A.S., Hungin A.P.S., Wooff D., Childs S. (2004). The effect of *Helicobacter pylori* and its eradication on gastro-oesophageal reflux disease in patients with duodenal ulcers or reflux oesophagitis. Aliment. Pharmacol. Ther..

[40] Haenszel W., Mantel N. (1959). Statistical Aspects of the Analysis of Data from Retrospective Studies of Disease. J. Natl. Cancer Inst..

[41] Berlin J.A., Laird N.M., Sacks H.S., Chalmers T.C. (1989). A comparison of statistical methods for combining event rates from clinical trials. Stat. Med..

[42] DerSimonian R., Laird N.M. (2015). Meta-analysis in clinical trials revisited. Contemp. Clin. Trials.

[43] Uotani T., Sugimoto M., Nishino M., Kodaira C., Yamade M., Sahara S., Yamada T., Osawa S., Sugimoto K., Tanaka T. (2012). Ability of Rabeprazole to Prevent Gastric Mucosal Damage From Clopidogrel and Low Doses of Aspirin Depends on CYP2C19 Genotype. Clin. Gastroenterol. Hepatol..

[44] Sugimoto M., Furuta T., Shirai N., Kajimura M., Hishida A., Sakurai M., Ohashi K., Ishizaki T. (2004). Different dosage regimens of rabeprazole for nocturnal gastric acid inhibition in relation to cytochrome P450 2C19 genotype status. Clin. Pharmacol. Ther..

[45] Furuta T., El–Omar E.M., Xiao F., Shirai N., Takashima M., Sugimurra H. (2002). Interleukin 1β polymorphisms increase risk of hypochlorhydria and atrophic gastritis and reduce risk of duodenal ulcer recurrence in Japan. Gastroenterology.

[46] Sugimoto M., Ohno T., Yamaoka Y. (2011). Expression of angiotensin II type 1 and type 2 receptor mRNAs in the gastric mucosa of *Helicobacter pylori*-infected Mongolian gerbils. J. Gastroenterol..

[47] Wolfe M., Nompleggi D. (1992). Cytokine inhibition of gastric acid secretion—A little goes a long way. Gastroenterology.

[48] El–Omar E.M., Penman I.D., Ardill J.E., Chittajallu R.S., Howie C., McColl K.E. (1995). *Helicobacter pylori* infection and abnormalities of acid secretion in patients with duodenal ulcer disease. Gastroenterology.

[49] Koike T., Ohara S., Sekine H., Iijima K., Kato K., Toyota T., Shimosegawa T. (2001). Increased gastric acid secretion after *Helicobacter pylori* eradication may be a factor for developing reflux oesophagitis. Aliment. Pharmacol. Ther..

[50] Hamada H., Haruma K., Mihara M., Kamada T., Yoshihara M., Sumii K., Kajiyama G., Kawanishi M. (2000). High incidence of reflux oesophagitis after eradication therapy for *Helicobacter pylori*: Impacts of hiatal hernia and corpus gastritis. Aliment. Pharmacol. Ther..

[51] Yamaoka Y., Orito E., Mizokami M., Gutiérrez Ó., Saitou N., Kodama T., Osato M.S., Kim J.G., Ramirez F.C., Mahachai V. (2002). *Helicobacter pylori* in North and South America before Columbus. FEBS Lett..

